# News and Coming Events

**Published:** 1908-05-23

**Authors:** 


### NEWS AND COMING EVENTS.

The King has signified his intention of laying the commemoration stone of King Edward VII.'s Hospital and Dispensary for Windsor and District, now being built on a plot of Crown land opposite Combermere Barracks, Windsor.

Dr. A. H. Hogarth, M.B., B.Ch. Oxon., D.P.H., has been appointed medical officer of health to the Buckinghamshire County Council.

M. Henri Dunant, founder of the Red Cross Association and initiator of the Geneva Convention, celebrated his 80th birthday on Friday, May 8.

Mr. Newton H. Nixon, Secretary of University College Hospital, has retired, through ill-health, and has been granted a pension of £300 a year.

Mr. John Poland, F.R.C.S., has resigned the post of senior surgeon to the Miller Hospital, Greenwich, with which he has been connected for many years.

Dr. Lillias Hamilton, who was at one time in charge of the Dufferin Hospital, Calcutta, has succeeded Miss Faithfull as warden of the Studley Horticultural College for Women.

Dr. George Reid medical officer of health for Staffordshire, has been appointed by the Home Secretary to be a member of the committee to inquire into the use of lead in the manufacture of earthenware and china.

Mr. Rickman J. Godlee, F.R.C.S., Vice-President of the Royal College of Surgeons, England, has been elected the representative of that college on the Council of University College, Bristol, in succession to Mr. Thomas Bryant, resigned.

A marble bust of the late Mr. George Herring, the munificent benefactor to the Hospital Sunday Fund, will be unveiled at the Mansion House on Monday, June 15. The bust is a gift to the Corporation, and will remain permanently in the saloon.

Colonel W. W. Chapman, of Manchester, has paid into the bank the sum of £5,000 as a personal contribution to the funds of the Manchester and Salford Hospitals, the only condition accompanying it being that it shall be applied exclusively to hospital purposes.

To avoid misconception the committee of the Kensington Dispensary and Children's Hospital, 49 Church Street, Kensington, wish it to be known that this institution has no connection whatever with the Kensington General Hospital, Richmond Road, Earl's Court.

The annual dinner in aid of the funds of the City of London Hospital for Diseases of the Chest was held on May 12 at the Trocadero Restaurant, Mr. Herbert Robertson, chairman of the committee, presiding. In proposing the toast of the evening, "Prosperity to the Hospital," the chairman said it was 60 years since the hospital began its work in Liverpool Street, and during that time 240,137 in-patients and 737,978 out-patients had been treated. Large numbers of poor patients could not be admitted owing to want of funds. More than 40,000 persons died each year of consumption in Great Britain and Ireland, and of those more than 8,000 fatal cases occurred in London alone. If the wards now closed for want of funds could be re-opened at that institution, 200 more patients could be admitted annually.

The Committee of the Throat Hospital, Golden Square, London, have received a donation of £250 from Mr. Charles J. Heath, F.R.C.S., one of the surgeons of the hospital, for the purpose of increasing the number of beds in the hospital, and thus reducing delays in the performance of operations and the consequent suffering of poor patients.

Mr. Francis Fremantle, F.R.C.S., M.R.C.P., has been appointed whole-time county medical officer by the Hertfordshire County Council to carry out, as to one-half of his time his public health duties as hitherto, and as to the remainder of his time to superintend a system of medical inspection in schools by the 14 district medical officers of health and two other local school medical officers.

The statue of the Queen, recently erected in the grounds of the London Hospital, is to be unveiled by the Earl of Crewe on July 10. The ceremony will be performed in conjunction with the annual gathering of the hospital governors and committee at which the prizes awarded among the students and nurses of the hospital during the year will be presented.

Dr. F. R. Russell, one of the best-known medical practitioners in Surrey, died recently, after a brief illness, at Guildford, at the age of 55. He was a Justice of the Peace for the borough of Guildford, a member of the Town Council, and of the Joint Hospital Board. Dr. Russell was deeply interested in the Volunteer movement, and held the rank of lieutenant-colonel at the time of his death.

The Earl of Shaftesbury presided at the festival dinner held to increase the rebuilding fund of the Hospital for Women, Soho Square. The Chairman, in making an appeal for the hospital, said that the institution had reached its sixty-fifth year of existence and was worthy of the full measure of public support. The number of in-patients was double what it was thirty years ago. The rebuilding of the hospital will cost about £25,000, and about £15,000 has still to be subscribed.

At the last meeting of the Council of the Royal College of Surgeons of England, a letter was read from Professor Howard Marsh resigning his seat on the Council. The resignation was accepted with regret, and the president stated that the vacancy thus occasioned would be filled up at the annual meeting of the Fellows in July. The President reported that under the powers conferred on him by the Statutes of the University of Wales he had appointed Mr. John H. Morgan, C.V.O., F.R.C.S., a member of the Medical Board of the University.

The fortieth annual dinner in aid of the funds of the French Hospital and Dispensary took place on May 16 at the Hôtel Cecil. The French Ambassador, who was accompanied by Count de Manneville, the First Secretary, and several other members of the Embassy and the French Consul-General, occupied the chair, and the Lord Mayor and sheriffs were the principal guests. The Chairman, in proposing the health of the President of the French Republic, said M. Fallières would be in London in a few days and would visit the Hospital. The institution was founded in 1867 for the benefit of distressed foreigners of all nations requiring medical relief. Donations and subscriptions were announced amounting to £4,300.

The Duchess of Westminster will present the diplomas, medals, and certificates of the National Health Society at 5 p.m. on Wednesday, June 3, at Grosvenor House. The Earl of Derby, K.G., will be in the chair; and Lord Alverstone, Dr. Baxter Forman, Professor Kenwood, and Professor Sims Woodhead are announced to speak.

The annual general meeting of the Asylum Workers' Association (founded in 1895 to improve the status of asylum nurses and attendants, to provide for them "Homes of Rest and Nursing," and to enlist public sympathy in their work) will be held at 11 Chandos Street, Cavendish Square, W., on Friday, May 29, at 3.30 p.m., Sir William J. Collins, M.D., F.R.C.S., M.P., President of the Association, in the chair. Dr. Robert Jones, F.R.C.P., will give an address.

The Royal College of Surgeons in Ireland and its Medical School have issued a statement to the effect that both will be extinguished by the competition of a new State-aided medical school when the Irish Universities Bill becomes law. Reasons are given why the school should not be destroyed, and the statement, which is signed by the President, the Vice-President, and the Secretary, concludes by demanding from Parliament "that the School of Medicine of the Royal College of Surgeons shall receive treatment equivalent to that which is to be given to the Catholic University School of Medicine in Cecilia Street, which is to be the Medical School of the Dublin College of the new University."

The Duke of Abercorn, President of the West London Hospital, occupied the chair at the festival dinner held on May 19 at the Trocadero Restaurant. He pointed out that the West London Hospital ranked among the 16 largest hospitals in London. It relieved annually some 40,000 out-patients and 2,500 in-patients. Its work was carried on in a most needy district, and was of unfailing value. Unfortunately the accommodation was inadequate, and funds were urgently needed to erect new buildings. The main object of the dinner was to obtain funds to enlarge the nurses' quarters and to provide better accommodation for out-patients.

According to a Reuter's telegram an epidemic of recurrent typhoid fever has broken out at Moscow. The hospitals are overflowing, 2,000 patients being already under treatment, while 75 to 100 fresh cases occur daily. The municipality has asked the military authorities to transform the summer barracks into a typhoid hospital. A telegram from Kieff to the *Russ* states that at the Berditcheff prison, where 700 persons are confined, although there is only proper accommodation for 250, there are over 100 cases of typhoid. The majority of the patients lie on straw on the floor, there being neither mattresses nor linen.

At the annual meeting of the Association of Medical Men Receiving Resident Patients, presided over by Dr. David Walsh, Dr. Hubert Biss, the honorary secretary, reported considerable progress in the work of the Association. The transference of the office from Bournemouth to 55 Outer Temple, Strand, W.C., has been found advantageous. The amount payable by new members for the first year has been reduced to one guinea, the subscription for subsequent years remaining at five shilings. The principle that no member should take less than three guineas a week for a patient save under exceptional circumstances was reaffirmed by this meeting.

The annual meeting of the Convalescent Homes Association will be held at 32 Sackville Street, W., on Thursday, May 28, at 4.30 p.m., Sir William S. Church, Bart., K.C.B., in the chair.

Princess Henry of Battenberg visited St. Mary's Hospital, Paddington, on the afternoon of May 19 and opened a three days' fête and bazaar in the Clarence Memorial Wing, in aid of the funds of that institution. Princess Henry was received by Mr. H. A. Harben, Chairman of the Hospital, Lady Dimsdale, President of the fête, Mrs. G. A. Byron, Hon. Secretary, and the Reception Committee, the members of which were presented to her Royal Highness, who was afterwards conducted to the out-patients' hall, where a bouquet was handed to her by Miss M. Harben. After declaring the fête open, Princess Henry inspected the stalls, where she made a number of purchases. Her Royal Highness subsequently visited Sir Almroth Wright's bacteriological laboratory in the inoculation department of the hospital. The fête was re-opened on Wednesday afternoon by the Lord Mayor and Lady Mayoress, and on Thursday afternoon by Lady de Grey.

At the annual festival dinner of King's College Hospital, held at the Hôtel Cecil on May 11, the American Ambassador, the Hon Whitelaw Reid, who presided, delivered an eloquent appeal for increased financial support for the hospital. The rebuilding of the hospital will cost £300,000, of which £194,000 has been received; and in addition to the large sum still required for this purpose money is needed to keep the existing hospital going. Mr. Whitelaw Reid drew attention to the great benefits which the hospital has conferred upon humanity, and enlarged on the importance of maintaining the voluntary character of hospital support in this country. He appealed for the continuance of the personal relation between the giver of charity and the sick poor who receive it, which would be abolished by the intervention of the State. The Hon. W. F. D. Smith, M.P., who replied, stated that the first contracts for the new building were let, and thenceforward until the first 360 beds were ready work would, he trusted, steadily progress.

